# Implantable Cardioverter Defibrillator in Primary and Secondary Prevention of SCD—What We Still Don′t Know

**DOI:** 10.3390/jcdd9040120

**Published:** 2022-04-16

**Authors:** Andreea Maria Ursaru, Antoniu Octavian Petris, Irina Iuliana Costache, Ana Nicolae, Adrian Crisan, Nicolae Dan Tesloianu

**Affiliations:** 1 Department of Cardiology, Emergency Clinical Hospital “Sf. Spiridon”, 700111 Iasi, Romania; andreea_ursaru@umfiasi.ro (A.M.U.); ii.costache@yahoo.com (I.I.C.); ana_nicolae@umfiasi.ro (A.N.); crisanadrian93@yahoo.com (A.C.); dan.tesloianu@umfiasi.ro (N.D.T.); 2 Department of Cardiology, “Grigore. T. Popa” University of Medicine and Pharmacy, 700111 Iasi, Romania

**Keywords:** implantable cardioverter defibrillator, primary prevention, secondary prevention, ischemic cardiomyopathy, non-ischemic cardiomyopathy, early ICD implantation, myocardial infarction, sudden cardiac death

## Abstract

Implantable cardioverter defibrillators (ICDs) are the cornerstone of primary and secondary prevention of sudden cardiac death (SCD) all around the globe. In almost 40 years of technological advances and multiple clinical trials, there has been a continuous increase in the implantation rate. The purpose of this review is to highlight the grey areas related to actual ICD recommendations, focusing specifically on the primary prevention of SCD. We will discuss the still-existing controversies strongly reflected in the differences between the international guidelines regarding ICD indication class in non-ischemic cardiomyopathy, and also address the question of early implantation after myocardial infarction in the absence of clear protocols for patients at high risk of life-threatening arrhythmias. Correlating the insufficient data in the literature for 40-day waiting times with the increased risk of SCD in the first month after myocardial infarction, we review the pros and cons of early ICD implantation.

## 1. Introduction

Cardiovascular diseases account for approximately 18.6 million deaths per year worldwide, 25% of which are sudden cardiac deaths (SCD) [1]. According to other statistics published in 2018 from the American Heart Association (AHA), cardiovascular disease causes over 850,000 deaths in the United States yearly [2,3,4,5]. Apart from these sad numbers, SCD continues to pose a significant challenge despite significant progress in the cardiovascular field in the last decades. The majority of SCDs in youths are related to cardiomyopathies, myocarditis, channelopathies and substance abuse, while in the elderly, chronic cardiac pathologies such as coronary artery disease, valvulopathies and heart failure (HF) are more often the cause [6]. Therefore, the identification of patients at high risk for SCD that would benefit from an implantable cardioverter defibrillator (ICD) has gained paramount importance [7]. Regarding the etiopathogeny of SCDs, almost 90% are related to electrical mechanisms, the most frequent of which are ventricular fibrillation (VF) and ventricular tachycardia (VT) [7,8,9]. From the 1980s, the natural history of SCD drastically changed: from a non-programmable device able only to recognize and treat VF [10] to capabilities such as antitachycardia pacing [ATP], low-energy synchronized cardioversion or biventricular pacing for HF treatment and subcutaneous ICDs [11,12].

In this paper we will discuss the main trials that influenced the ICD implant indications in the current worldwide guidelines. Performing a closer and critical analysis, revealing the unspoken results of individual controversial studies that grounded the guidelines’ recommendations and correlating them with the help of metanalysis, highlights the need to reconsider and reformulate these directions.

## 2. How to Identify the Patients in Need of an ICD—What do the Guidelines Say?

The first step for successful ICD therapy is the appropriate selection of patients. The guidelines in use differentiate between two major categories: primary and secondary prevention of SCD. Whereas secondary prevention is defined by patients who have experienced a symptomatic life-threatening sustained VT or VF, primary prevention is a useful tool for patients at an increased risk for such an event [12,13,14,15,16]. Reduced left ventricular ejection fraction (LVEF) is the most used parameter associated worldwide with all-cause and cardiovascular mortality and, at the same time, the variable that indicates ICD placement in the primary prevention of SCD. MADIT II (Second Multicenter Automated Defibrillator Implantation Trial II) and SCD HeFT (Sudden Cardiac Death in Heart Failure Trial), two landmark trials that proved the efficacy of ICD therapy in subjects with severely depressed left ventricular systolic function, showed similar survival benefits in ischemic and non-ischemic patients receiving an ICD [17,18]. These two trials represent the foundation of guidelines and recommendations across different geographic regions regarding the primary prevention of SCD in reduced ejection fraction heart failure [6,19,20]. According to the 2016 European Society of Cardiology (ESC) Heart Failure Guidelines, the patients with a life expectancy longer than 1 year and good functional status had a IA class recommendation for ICD placement if there was ischemic preconditioning (unless the subjects have had a myocardial infarction in the prior 40 days) and a IB indication in patients with a non-ischemic etiology of the symptomatic HF [13]. The new 2021 ESC Guidelines maintain the indications regarding the secondary prevention of SCD, specifying with a class I level of evidence A, that subjects who have recovered from ventricular arrhythmias (VAs) causing hemodynamic instability, without reversible causes and after more than 48 h since a myocardial infarction (MI), must receive an ICD—a constant statement when compared to the 2016 recommendations [12]. The situation is different concerning the indication class in the primary prevention of SCD: while patients with ischemic HF have a constant IA recommendation class for ICD placement, in patients with symptomatic systolic non-ischemic CMP, the recommendation was downgraded from a IB recommendation class in the 2016 ESC HF guidelines to a IIaA recommendation in 2021 [12,13]. The American Heart Association Guidelines make no differentiation between patients with ischemic and non-ischemic CMP, both categories having a class I level of evidence A recommendation for ICD implantation [14]. Canadian guidelines [15] also maintained no differentiation between ischemic and non-ischemic heart failure by indicating an ICD with a strong recommendation and high quality of evidence. Finally, the Australian guidelines published in August 2018 downgraded their recommendation on ICDs in non-ischemic cardiomyopathy to a weaker recommendation and lower level of evidence [16], but there were authors who contested this change [21,22]. The discrepancies between the international guidelines for the primary prevention of SCD can be analyzed in Table 1. In the authors’ opinion, there is little evidence supporting the recent downgrading of the ICD placement indication in patients with symptomatic systolic non-ischemic heart failure. The major differences between guidelines arise from the opposite results of the trials on which each of these are based. Reviewing the existing literature by including available metanalyses would offer a broader view of ICD’s advantages in non-ischemic primary prevention patients, when comparing it with the outcomes of single studies.

## 3. The Unanimity of Secondary Prevention

While there are multiple debates and differences between guidelines’ recommendations worldwide for the primary prevention of SCD, it appears that an agreement was reached regarding secondary prevention. Currently there is a consensus that patients with sudden cardiac arrest have a significantly increased risk of recurrences and require an ICD. Multiple studies have shown all anti-arrhythmic drugs have failed to improve survival in comparison with defibrillation therapy. One of the most important trials comparing ICD therapy with antiarrhythmics, the Anti-arrhythmics Versus Implantable Defibrillators (AVID) trial randomized subjects with prior cardioversion for sustained VT or resuscitated VF to ICD placement or class III anti-arrhythmics, primarily amiodarone [23]. In the ICD group, after 3 years follow-up a significant reduction in the primary end point of mortality was found, with a predominant benefit in those with reduced LVEF. The Canadian Implantable Defibrillator Study (CIDS) included patients with resuscitated VT/VF or unmonitored syncope, also undergoing either ICD or amiodarone therapy. An important, but still non-significant decrease in the overall and arrhythmic mortality was observed in the ICD group [24]. Similar to CIDS, the Cardiac Arrest Study Hamburg (CASH) randomized patients with prior sudden cardiac arrest, comparing ICD therapy to amiodarone/metoprolol, and the singular result showed no differences: a non-significant reduction in the overall mortality in the ICD group [25]. The metanalyses of these studies led to a unitary conclusion which was different from the results of each study presented separately: a statistically significant decrease in the overall and arrhythmic mortality with ICD therapy [26,27]. This is why study results should not be taken ad litteram, but rather integrated into the literature and interpreted by correlation with other studies in the same field. Although CIDS and CASH did not demonstrate a statistically significant reduction in mortality in ICD therapy, together with the results from AVID, they represent the cornerstone of ICD indications in today′s guidelines for the secondary prevention of SCD. 

Despite actual recommendations to exclude patients with transient or reversible causes of VAs (acute ischemia, recent MI, drug overdose, severe dyselectrolytemias), the AVID registry revealed that patients in this category who did not benefit from ICD placement had a remarkably high subsequent mortality [23]. Thereby, ICD therapy may also be considered in some subjects with an apparently correctable underlying cause of the arrhythmia.

## 4. Primary Prevention: Ischemic Cardiomyopathy (ICMP) versus Non-Ischemic Cardiomyopathy (NICMP)

Identifying patients at high risk and treating them prophylactically with ICD therapy is a current challenge, yet there were multiple studies that have helped in the purpose of establishing the role of primary prevention ICD both in ischemic and non-ischemic cardiomyopathies.

The 2003 Multicenter Unsustained Tachycardia Trial (MUSTT) was one of the first trials that included ischemic patients with LVEF < = 40% and non-sustained VT. From the subjects with inducible VT at electro-physiology (EP) study, 353 were randomized to the conservative group and 351 to the antiarrhythmic therapy group, further divided into three groups: 45% of them receiving antiarrhythmic drugs, 46% ICD, and 7% no therapy. The trial demonstrated a survival benefit only in ICD recipients, while anti-arrhythmic drugs did not reduce the risk of arrhythmic death [28]. In a similar manner, the Multicenter Automatic Defibrillator Implantation Trial (MADIT) included subjects with MI, reduced ejection fraction (≤35%), non-sustained VT and inducible VT at EP study not suppressible with intravenous procainamide. They were randomized into ICD or conventional drug therapy recipients (74% of subjects): amiodarone, digitalis, sotalol, disopyramide, mexiletine, procainamide, tocainide and beta-blockers. The reduction in all-cause mortality in the ICD group was of 54% when compared to the best conventional treatment (p-0.009) [29].

The 2010 MADIT II studied ischemic patients with an LVEF ≤ 30% without conditioning inclusion by criteria such as inducible arrhythmia at EP study or NSVT, thus allowing the evaluation of a much broader population (1232 subjects). There were two arms–ICD versus conventional medical therapy–a 31% relative reduction (hazard ratio 0.69) was observed in the ICD arm and the study stopped early. After 8 years of long-term follow-up, the ICD arm still had lower total mortality: 49% vs. 62% (hazard ratio 0.66) [17]. 

The Sudden Cardiac Death in Heart Failure study (SCD-HeFT) was the largest study in the field of primary prevention, comprised of 2.521 symptomatic HF patients with ischemic or non-ischemic substrate and LVEF ≤ 35%; 829 patients were randomized to the ICD arm, 845 to amiodarone and 847 to placebo, with a median follow-up time of 45.5 months. While placebo and amiodarone were not associated with a decreased risk of death, the ICD therapy reduced mortality by 23%. After 6 years, a decrease in mortality was maintained by 7.2%. There was no difference in benefit between ischemic and non-ischemic patients [18].

There are a few studies in the literature that focused only on NICMP: Amiodarone versus Implantable Defibrillator (AMIOVIRT), the Cardiomyopathy Trial (CAT) and Defibrillators in Non-Ischemic Cardiomyopathy Treatment Evaluation (DEFINITE) [30,31,32]. The CAT [30] was designed to randomize 1348 patients with an expected mortality rate of 30% at a 1-year follow-up, but it recruited no more than 104 patients with a 5.6% mortality rate. As a result of these preliminary outcomes, the study was terminated prematurely. The DEFINITE trial [32], the first randomized trial of primary prevention in NICMP, included 458 patients and reported 68 deaths: 28 patients (8.1%) in the ICD group and 40 patients (13.8%) in the standard medical care group [hazard radio (HR)–0.65; 95% confidence interval (CI) 0.40–1.06; p-0.08]—close to the overall mortality target of 15% in the standard care arm and 7.5% in the ICD arm, but also not enough to reach it. While failing to achieve the primary endpoint, the results of this study proved instead the true purpose of ICD therapy, i.e., the reduction in arrhythmic SCD, which was outreached: 3 deaths in the ICD patients compared to 14 in the standard medical therapy patients (HR-0.20; 95% CI 0.06–0.71; p-0.006). The significant reduction in mortality that comes with ICD therapy in non-ischemic patients was accredited by two metanalyses [33,34]. Theuns et al. in their 2010 work concluded that the benefit in all-cause mortality is similar in patients with ischemic heart disease patients (relative risk 0.67; 95% CI 0.51–0.88) when compared to non-ischemic cardiomyopathy (relative risk 0.74; 95% CI 0.59–0.93) [34]. The results of all the presented analyzes, with the main role attributed to the SCD-HeFT trial, led to the establishing of the role of ICD in NICMP patients. 

Since the publication of all these trials, certain guidelines’ recommendations have changed, mostly influenced by the publication of the DANISH trial [35]. The DANISH trial included only patients with NICMP: 556 patients in the ICD arm and 560 patients who received standard optimal medical therapy. Although the risk of SCD was halved, the investigators of the study concluded that the implantation of an ICD in non-ischemic patients did not provide an overall survival benefit. A following analysis revealed that ICD implantation was associated with reduced all-cause mortality in patients ≤70 years of age [36] and that the benefit of implantation decreased with older age, given the fact that older patients were more likely to die of other causes rather than SCD compared to the younger population. 

The primary end-point of the DANISH trial, overall mortality, was slightly reduced (HR-0.87 with 95% CI: 0.68–1.12) without reaching statistical significance (p-0.28). Despite its neutrality in decreasing global mortality, the DANISH trial greatly reduced (HR–0.50 with 95% CI: 0.31–0.82) the SCD risk, with a result that was statistically significant (p–0.005). Although the trial’s power was calculated on reducing all-cause mortality, the results only imply the intuitive fact that a cardiac defibrillator can only reduce arrhythmic SCD and does not majorly influence non-cardiac or pump failure deaths. In the DANISH trial, death occurred in 120 patients (21.6%) in the ICD arm and in 131 patients (23.4%) in the standard medical therapy arm. Of the 131 deaths, only 46 of them (one-fourth lower than initially presumed) were SCDs. Owing to the clear specificity of an ICD in preventing arrhythmic death, it is arguable that the investigators were testing a therapy in a population with too few treatable events (arrhythmic deaths) and a much larger number of untreatable events (non-cardiovascular deaths and non-sudden cardiac death) in order to demonstrate the effectiveness of the treatment. It is also to be mentioned that in the Kaplan–Meier graph the hazard assumption was found to be violated with crossover after the reduced rate of death in the ICD population, results that emphasize the idea that the benefit with respect to mortality of an ICD depends on time [21]. Moreover, several metanalyses that included the DANISH trial showed that the reduction in all-cause mortality in patients who benefited from an ICD is identical in those with HFrEF due to ischemic and non-ischemic causes (HR–0.76) [21]. Beggs et al. in their 2017 analysis, which examined the effect of ICDs in NICMP, found an overall survival benefit, but the effect was significantly weakened by the inclusion of the DANISH trial [37]. 

Another important aspect is that in the DANISH trial 58% of the patients assigned to the standard clinical care (control) group received a CRT-P device. The increased number of CRT-P recipients raises the question whether we can extrapolate the data to patients with NICMP that do not benefit from resynchronization therapy. Numerous trials have shown CRT pacing alone reduces mortality in NICMP patients when compared to medical therapy: in heart failure trials 40% of all deaths are sudden, and the percentage increases proportionally with the total mortality; 50% to 60% of these SCDs are preventable with the addition of an ICD [38,39]. In the CRT-P trials, sudden death is lowered to only 23–32%; in the DANISH study it was only 35% of all deaths. Even CRT-eligible patients who did not receive CRT have a much lower proportion of sudden death, in the 25% range, influenced by the mode of death in advanced HF [38,40,41,42]. Furthermore, metanalysis of multiple trials, including DANISH, showed a clear benefit of ICD implantation on overall survival for non-CRT NICMP patients and this effect was even more prominent in patients pooled from the DANISH group. We may comment on the fact that in patients older than 70 years old the CRT proportion was 68% and, probably not as a simple coincidence, ICD implantation was associated with reduced all-cause mortality only in patients ≤70 years of age; adding the results from the population older than 70 years, the authors concluded that ICD shows no benefits in NICMP because it did not reduce long-term global mortality compared to standard medical care. It must be remembered that CRT does not represent “standard care” in every patient with HF; resynchronization therapy improves HF symptoms and prognosis, induces ventricular remodeling and reduces the rate of onset of VAs detected by ICDs in patients implanted for primary prevention [43,44,45]. The large proportion of patients with CRT in the DANISH trial diminishes the chance of observing effects of ICD, so this may be considered an a priori limitation of the study and one of the main reasons why we should not extrapolate the results of this controversial study to the entire NICMP population [46,47]

All of the above may raise some important questions regarding the use of ICD in NICMP patients. Therefore, it would be more appropriate to ground guidelines’ recommendations on solid evidence, such as the results of metanalyses and not on individual studies, as is the case for the DANISH trial and the ESC 2021 HF guidelines. In an analysis of over 40,000 patients from 12 HF trials, rates of SCD decreased by 44% over the last 20-years. This is explained by the advances in HF treatment: beta-blockers, mineralocorticoid receptor antagonists, angiotensin receptor-neprilysin inhibitor and cardiac resynchronization therapy added to the benefits of the cardioverter defibrillator therapy, all of these reducing the risk of sudden death [48]. For instance, the 2015 PARADIGM-HF study, which included 8399 patients with chronic heart failure and left ventricular ejection fraction ≤40%, showed the angiotensin-receptor-neprilysin inhibitor was superior to enalapril in reducing sudden cardiac death [41]. There are multiple studies [49,50,51] that prove comparable rates of ICD efficacy in ischemic and non-ischemic patients: one example is an analysis of 387 consecutive ICD recipients that found NICMP was associated with even higher rates of recurrent VT/VF and appropriate ICD therapies in primary prevention patients when compared to ICMP [49], which were confirmed in a retrospective study by Verhagen et al. demonstrating similar mortality rates in ICMP and NICMP [51]. Additionally, in the same order of ideas, metanalyses including data from all studies over the past 20 years in ICD primary prevention, including the DANISH trial, have shown a significant reduction in global mortality associated with ICD use in NICMP [47,52]. Another important aspect is that NICMP encompasses a diverse range of etiologies, including dilated cardiomyopathy, infiltrative, inflammatory, neuromuscular, alcohol and drug toxicities whereby the prognosis differs widely as do the management strategies [21,53]. A further noteworthy standpoint is the impact of transvenous ICD (TV-ICD)-related complications on cardiovascular and all-cause mortality, considering the high rate of device-related issues reported in the above-mentioned studies. Subcutaneous ICDs (s-ICDs) were specifically designed to overcome some specific TV-ICD complications such as lead dislodgement, lead failure/fracture, pneumothorax, cardiac perforation, venous occlusion or systemic infection. A large study on 1254 patients [54] noted similar s-ICD and TV-ICD complication rates, with the major difference being the lack of device-related deaths in the s-ICD group. The most common s-ICD incidents were represented by pocket hematoma and unanticipated generator replacement. Other issues mentioned were lead fractures or lead tip erosions [55], rare events that can be successfully managed by straightforward extraction and reimplantation, in contrast with TV-ICD infections or lead-related complications which might result in endocarditis or lead extraction associated with increased mortality rates. Avoiding life-threating TV-ICD complications could potentially decrease the morbidity and mortality in ICD patients and impact the results of future studies that need to be performed in s-ICD recipients.

## 5. The First 40 Days after MI—A Gray Area: Early ICD for Primary Prevention of SCD?

While most studies have expanded the role of ICD therapy in clinical practice, ICD therapy in selected populations has demonstrated an apparent lack of efficacy, results that have influenced the current guidelines: The Defibrillator in Acute Myocardial Infarction (DINAMIT) [56] trial was a randomized open-labeled comparison of ICD therapy (in 332 patients) and non-ICD therapy (in 342 patients) in the first 6 to 40 days after a myocardial infarction. Patients enrolled in this study had LVEF ≤ 35% and impaired cardiac autonomic function defined as depressed heart-rate variability or an elevated average 24-h heart rate on continuous ECG monitoring. The primary outcome, all-cause mortality, was not different in the two groups (HR-1.08; CI 0.76–1.55; p-0.66), although there were 12 deaths due to arrhythmia in the ICD group as compared to 29 in the control group (HR-0.42; 95% CI 0.22–0.83; p-0.009) which shows a statistically significant reduction in arrhythmic deaths. It should be mentioned that the number of primary angioplasties was relatively low—only 27%—and the fact that the authors did not report data regarding the aldosterone receptor blocker therapy despite their proven role in reducing mortality among subjects with recent MI and LVEF ≤ 40%. Early prophylactic ICD implantation reduced the rate of arrhythmic death in the DINAMIT trial, so we can consider that ICD was a successful therapeutic option. 

The Beta-blocker Strategy Plus ICD (BEST) [57] trial enrolled 143 patients 5 to 30 days after an MI, with a mean LVEF of 31%. The authors noticed a trend of lower arrhythmic and all-cause mortality in favor of the ICD group versus conventional therapy, without reaching statistical significance. In the BEST + ICD study, the investigators found that the overall mortality of survivors of an acute MI remained high (16% at 1 year and 24% at 2 years) despite optimal medical therapy, which indicated the enrolment of a high-risk subgroup of patients who deserve implementation of efficient preventive measures.

ICD implantation after MI was also evaluated by the Immediate Risk Stratification Improves Survival (IRIS) [58] study, a randomized, prospective, open-label, multicentric trial in which patients 5–31 days after an MI with LVEF ≤ 40%, heart rate greater than 90 beats per minute (bpm) or non-sustained VT on continuous ECG monitoring were randomized to ICD (445 patients) or standard medical therapy (453 patients). During a mean follow-up of 37 months, the primary endpoint (overall mortality) was the same in the two groups (HR-1.04; 95% CI 0.81–1.35; p-0.78) but there were fewer SCDs and a higher number of non SCDs in the ICD group than in the control group. Major differences in baseline characteristics between the two groups, the different response to HF treatment and the substrate of acute MI, could explain an increase in death due to causes other than SCD. ICD significantly reduced the rate of SCD (HR 0.55), similar to DEFINITE trial. Since ventricular arrhythmias are the most common cause of SCD [59,60] these studies have proven useful from this point of view, even though their results are cited as evidence against early ICD prevention after MI. 

Elayi et al. [61] in their 2017 analysis expose different hypotheses for the lack of meaningful survival improvement in early ICD trials. An interesting supposition is the impact of defibrillation threshold (DFT) testing and sedation on the ongoing remodeling myocardium in the early recovery phase after MI. Considering that both IRIS and DINAMIT performed DFT testing in ICD patients, this could influence long-term myocardial remodeling and thus the primary outcome of these studies—all-cause mortality. DFT testing has been associated with an increase in different biomarkers, including those related to apoptosis. However, the large SIMPLE (cardioverter defibrillator implantation without induction of ventricular fibrillation: a single-blind, non-inferiority, randomized controlled) trial [62] showed no differentiation during the 3.1 years follow-up between patients with DFT versus no DFT during ICD implantation regarding all-cause mortality, despite a slight increase in periprocedural complications in the DFT arm. Nonetheless, neither SIMPLE nor other trials addressed DFT testing in early post-MI subjects, who may be the most exposed to adverse effects of myocardial remodeling [61].

The DAPA (Defibrillator After Primary Angioplasty) [63] trial is a more recent multicentric study which included patients between 30 and 60 days after MI, who underwent primary PCI. There was a significantly decreased rate of global mortality of 5% vs. 13% after 3 years follow-up and 18% vs. 38% after 9 years in the ICD group. The cardiac mortality was also significantly lower in the ICD group, with no differences regarding non-cardiac deaths. In contrast with the aforementioned trials, high rate therapy (high voltage electrical shock for VAs ≥ 190 bpm) and ATP was programmed. Using high-rate detection and delayed therapy, ATP and reducing the unnecessary pacing by avoiding rate responsive modes could additionally reduce mortality. The main characteristics of the four early ICD trials for primary prevention of SCD after acute MI (DINAMIT, BEST+, IRIS, DAPA) are presented in Table 2.

One of the landmark trials for acute ischemia, the VALsartan In Acute myocardial iNfarcTion (VALIANT) trial [64], that enrolled patients with an LVEF ≤ 40% after an acute MI, revealed that the risk of SCD is the highest in the first 30 days, in patients with an LVEF ≤ 30% (2.3% per month). 

Combing the analysis of the above-mentioned studies with the data from the 8453 patients in the Zoll registry which revealed that 75% of shocks in patients with wearable cardioverter-defibrillator (WCD) occurred in the first month after the MI [65], it seems logical to seek out an appropriate solution for the most vulnerable period—the first 40 days after the acute event. There is no clear recommendation to guide physicians in this period, the WCD having only a IIb class of indication in the ESC and ACC/AHA/HRS guidelines, seemingly being a rather unsuitable solution for patients with HF who are at risk of SCD. It is not by far a perfect alternative to an ICD because it lacks functions such as ATP and post-shock anti-bradycardia pacing, without counting the rate of inappropriate shocks and the reduced compliance of the patients [66].

Since there is no viable alternative available and different metanalyses of early ICD studies have shown a reduced mortality in implanted patients, this may be the only reserve for patients at high risk of SCD post MI; even considering early ICD, we retain the difficulty of choosing the appropriate candidates, which is a crucial step. A reduced ejection fraction remains the single best predictor of SCD and the main tool in identifying patients who need an ICD, although as a freestanding risk stratification tool, it has major limitations: a significant number of subjects with preserved or moderately reduced LVEF die due to SCD, and only approximately one-third of the ICD recipients will benefit from defibrillation therapy during the first battery life. A depressed EF is a predictor of both arrhythmic and non-arrhythmic cardiac mortality, such as progressive heart failure [67]. As LVEF decreases, the risk of non-sudden death increases, and while the absolute risk of SCD is high for a patient with an LVEF in the range of 15%, the proportional risk of SCD may be in reality lower because of the patient’s increased risk of death through pump failure. Although it may seem counterintuitive, subjects with an EF at the upper border of HFrEF may benefit more from device implantation than patients with an LVEF of approximately 15%, because their likelihood to die due to arrhythmia is higher even though they theoretically have a lower absolute risk of SCD [68]. Considering the aforementioned shortcomings of EF, variables as ventricular premature beats, QRS duration, signal-averaged ECG or programmed ventricular stimulation were studied as risk stratification factors for the decision on prophylactic ICD placement. These have proven inaccurate, the main disadvantage (similar to EF) being the limited sensitivity in predicting both sudden and non-sudden death. Therefore, if it is unrealistic that a single test will prove adequate, a combination of characteristics, as the large, ongoing European PROFID project [69] involves, may significantly improve risk stratification in the future. Multiple clinical prediction models [70,71], including a combination of variables for the estimation of the arrhythmic risk post-MI, could not be implemented into the daily practice mainly because enhancements in their predictive potential are necessary and their clinical utility must be proven [72]. 

In regard to the recovery of LVEF after acute MI, one study of 600 consecutive patients with MI treated with primary angioplasty showed a mean relative improvement of 6% from day 4 to 6 months after PCI [73]. Reibis et al. [74], which included 277 consecutive patients with LVEF ≤40% at approximately 1 month after acute MI confirmed the increase in LVEF by 6%. The authors’ affirmation that the improvement of LVEF is rather a result of interindividual variability, intraindividual variability and regression towards the mean (which reflects a statistical effect), with the slight addition of the actual increase in LV function, should be emphasized. Even after complete revascularization, the systolic dysfunction persists and it is rather improbable that the true LV function will return to normal in patients with a damaged ventricle, the recovery being usually modest, although this can only be expected in stunned or hibernating myocardial segments [75]. Returning to the question of whether we need to wait 40 days after MI to decide for an ICD, from the perspective of LVEF recovery, it is meaningless—due to time constraints, it is not worth waiting for the full recovery of the hibernating myocardium, which could take up to 14 months, as studies have shown [76]. Since the recovery from stunning occurs within 2 weeks, it is also worthless and risky to prolong the time to implant until 40 days post-MI [77,78]. 

There have been studies, such as the PREDICTS-Predicting Persistent Left Ventricular Dysfunction Following Myocardial Infarction: PREDiction of ICd Treatment study [79], that described a significant recovery of the LVEF after MI. The authors reported that 57% of the 231 patients with LVEF < 35% (mean EF at index event 28.1 ± 6.6) improved to >35% at 3 months follow-up post-MI. Whereas the majority of trials performed LVEF estimation at least a few days post-revascularization, PREDICTS assessed the ejection fraction 8 h “after the MI or percutaneous coronary intervention.” Thus, the increase in LVEF from the first 3 days after angioplasty, when the greatest improvement in regional systolic function occurs and when the stunned myocardium in smaller infarctions is partially recovered [80], was probably erroneously attributed to a long-term increase in the ejection fraction—3 months, making the results of the study arguable.

A systematic review, which included 76 observational studies and 12 randomized trials with more than 100,000 subjects, found that ICDs are effective in adults with a reduced ejection fraction and that the benefits extend beyond the trial populations [81]. ICD therapy conferred an additional benefit in mortality reduction, mainly by reducing the SCD by arrhythmia. Thereby, in patients with a history of myocardial infarction or heart failure, the ejection fraction should be the first parameter to be determined [82]. Furthermore, the 40 days waiting time after MI is random and it is due to fact that the two of the most important trials for the evaluation of ICDs in primary prevention, MADIT and MADIT II, included subjects only after three and four weeks, respectively, post acute MI. 

To summarize, correlating the increased risk of SCD in the first month post-MI and the insufficient data in the literature for 40-day waiting times with the fact that ICDs were associated with a lower risk of SCD in early ICD trials, the futility and risks that this delay brings with it become obvious, and choosing early ICD implantation may be the appropriate solution. We advocate the use of early ICD implantation, our standpoint being confirmed by a small retrospective analysis of 77 patients that brings supplementary arguments for the benefit of early ICD implantation in patients with a reduced ejection fraction after MI [83]. Of course, we still need larger, more representative trials and more multi-center registry data for confirmation.

## 6. Conclusions

This review explores in detail the concept of early ICD implantation and the efficiency of ICD in NICMP and reiterates the need for more accurate recommendations and for a pertinent and global understanding of the existing studies in the specialty literature. A few unanswered questions still remain: (i) Is one study enough to change current worldwide guidelines? (ii) Is it appropriate to modify the guidelines’ recommendations based only on trials and not on metanalyses, the latter being much more complete? (iii) What should the contraindication of implanting an ICD peri-MI be? (iv) Will it expose the patients with HFrEF to additional risks to wait 40 days after MI before implanting an ICD in the absence of solid evidence? All these dilemmas need proper answers soon and, furthermore, we should remember that integrating clinical judgement into the guidelines would be a trend many physicians would applaud. 

## Figures and Tables

**Table 1:** Discrepancies between international guidelines for primary prevention of SCD-classes of recommendation and levels of evidence.

Group of Patients	2016 ESC Guidelines	2021 ESC Guidelines	2013 ACCF/AHA Guideline for the Management of HF, 2017 ACC/AHA/HFSA Focused Update	2017 Canadian HF Guidelines	2018 Guidelines for the Prevention, Detection, and Management of HF in Australia
LVEF ≤ 35% despite ≥ 3 months of OMT, symptomatic HF (NYHA Class II–III), expected survival longer than 1 year • ischemic etiology (unless they have had an MI in the prior 40 days) • non-ischemic etiology	IA IB	IA IIa A	IA IA	Strong Recommendation, High Quality Evidence * Strong Recommendation, High Quality Evidence	Strong Recommendation FOR; Moderate Quality of Evidence ^†^ Weak Recommendation FOR; Low Quality of Evidence ^†^
LVEF 30%, at least 40 days post-MI, and NYHA class I symptoms while receiving OMT, with expected survival longer than 1 year	N/A	N/A	IB	Strong Recommendation, High Quality Evidence *	Strong recommendation FOR; high quality of evidence **

* at least 1 month post MI, and at least 3 months post coronary revascularization procedure; ** at least 1 month post MI; no mention of NYHA class; ^†^ no mention of NYHA class; HF = heart failure; ICD = implantable cardioverter-defibrillator; LVEF = left ventricular ejection fraction; MI = myocardial infarction; N/A = Not available; NYHA = New York Heart Association; OMT = optimal medical therapy.

**Table 2:** Early ICD implantation trials for primary prevention of SCD after acute MI.

Trial Name	DINAMIT	BEST+ ^†^	IRIS	DAPA ^†^
Year of publication	2004	2005	2009	2020
Year of enrollment	1998–2002	1998–2003	1999–2007	2004–2013
Inclusion criteria	Recent MI (6–40 days), LVEF ≤35% and one of the following: - RR intervals variability ≤70 ms on continuous ECG monitoring - Mean heart rate >80 beats per minute on continuous ECG monitoring	Recent MI (5–30 days), LVEF ≤35% and one or more of the following: - PVCs >= 10/h; - reduced HRV - positive SAECG + tolerance to metoprolol–at least 25 mg/day	Recent MI (5–31 days), LVEF ≤ 40% and one of the following: - HR ≥ 90 on ECG; - NSVT on continuous ECG monitoring	Recent MI (30–60 days)–Primary PCI for STEMI and ≥1 high risk factor: - LVEF <30% within 4 days - TIMI flow <3 after PCI - Primary VF - Killip class ≥2
No. of patients enrolled	674	138	898	262
No. of patients in ICD group/control group	332/342	79 (24)/59 *	445/453	129/133
Follow-up, mean	30 months +/− 13 months	540 days +/− 403 days	37 months (range 0–106)	36 months
ICD programming	VT: 175–200 bpm 16 intervals to detect (4 ATP sequences–burst) VF zone ≥200 bpm (18/24) VVI pacing mode 40 to 55 bpm	N/A	VT: 150–200 bpm (32 intervals to detect, no ATP) VF zone ≥200 bpm VVI pacing mode 40 bpm	Fast VT or VF ≥190 bpm (ATP burst during charging) VVI pacing mode/DDI mode with a long AV interval
DFT Testing	Yes	N/A	Yes	Yes
Reperfusion Therapy	66.5%	16.3%	86.8%	97%
All-cause mortality	62 in the ICD group 58 in the control group	13 in EPS/ICD group 13 in control group	116 in the ICD group 117 in the control group	40 patients (15%) 5% in the ICD group 13% in the control group
SCD	12 in the ICD group 29 in the control group	4 in EPS/ICD group 5 in the control group	27 in the ICD group 60 in the control group	3.1% in the ICD group 5.9% in the control group
Cardiac non-SCD	34 in the ICD group 20 in the control group	9 in EPS/ICD and control group	68 in the ICD group 39 in the control group	7.9% in the ICD group 16.1% in the control group
Total cardiac death	46 in the ICD group 49 in the control group	18 in EPS/ICD and control group	95 in the ICD group 99 in the control group	11% in the ICD group 22% in the control group

* In BEST+ 79 patients were randomized to EPS guided/ICD strategy, with only 24 inducible patients with ICD implantation; ^†^ BEST+ and DAPA trials were prematurely terminated because of a slow enrollment rate; AV = atrioventricular; DFT = defibrillation threshold; EPS = electrophysiological study; HR = heart rate; HRV = heart rate variability; ICD = implantable cardiac defibrillator; LVEF = left ventricular ejection fraction; MI = myocardial infarction; N/A = not available; NSVT = non-sustained ventricular tachycardia; PCI = percutaneous coronary intervention; PVCs = premature ventricular contractions; SAECG = signal-averaged electrocardiogram; SCD = sudden cardiac death; STEMI = ST-elevation myocardial infarction; VT = ventricular tachycardia; VF = ventricular fibrillation.
